# The Proliferation and Differentiation of Adipose-Derived Stem Cells in Neovascularization and Angiogenesis

**DOI:** 10.3390/ijms21113790

**Published:** 2020-05-27

**Authors:** Greg Hutchings, Krzysztof Janowicz, Lisa Moncrieff, Claudia Dompe, Ewa Strauss, Ievgeniia Kocherova, Mariusz J. Nawrocki, Łukasz Kruszyna, Grzegorz Wąsiatycz, Paweł Antosik, Jamil A. Shibli, Paul Mozdziak, Bartłomiej Perek, Zbigniew Krasiński, Bartosz Kempisty, Michał Nowicki

**Affiliations:** 1 The School of Medicine, Medical Sciences and Nutrition, University of Aberdeen, Aberdeen AB25 2ZD, UK; g.hutchings.16@abdn.ac.uk (G.H.); krzysztof.janowicz.16@abdn.ac.uk (K.J.); l.moncrieff.16@abdn.ac.uk (L.M.); 2 Department of Anatomy, Poznan University of Medical Sciences, 60-781 Poznan, Poland; kocherova.evgenia@gmail.com (I.K.); mjnawrocki@ump.edu.pl (M.J.N.); bkempisty@ump.edu.pl (B.K.); 3 Department of Histology and Embryology, Poznan University of Medical Sciences, 60-781 Poznan, Poland; mnowicki@ump.edu.pl; 4 Institute of Human Genetics, Polish Academy of Sciences, 60-479 Poznan, Poland; strauss@man.poznan.pl; 5 Department of Vascular, Endovascular Surgery, Angiology and Phlebology Poznan University of Medical Sciences, 61-701 Poznan, Poland; lukaszkruszyna@poczta.onet.pl (L.K.); zbigniew.krasinski@gmail.com (Z.K.); 6 Department of Veterinary Surgery, Institute of Veterinary Medicine, Nicolaus Copernicus University in Toruń, 87-100 Toruń, Poland; g.wasiatycz@umk.pl (G.W.); pantosik@umk.pl (P.A.); 7 Department of Periodontology and Oral Implantology, Dental Research Division, University of Guarulhos, São Paulo 07023-070, Brazil; jashibli@yahoo.com; 8 Physiology Graduate Program, North Carolina State University, Raleigh, NC 27695, USA; pemozdzi@ncsu.edu; 9 Department of Cardiac Surgery and Transplantology, Poznan University of Medical Sciences, 61-848 Poznań, Poland; bperek@ump.edu.pl; 10 Department of Obstetrics and Gynecology, University Hospital and Masaryk University, 601 77 Brno, Czech Republic

**Keywords:** adipose, stem, differentiation, vascularization, angiogenesis

## Abstract

Neovascularization and angiogenesis are vital processes in the repair of damaged tissue, creating new blood vessel networks and increasing oxygen and nutrient supply for regeneration. The importance of Adipose-derived Mesenchymal Stem Cells (ASCs) contained in the adipose tissue surrounding blood vessel networks to these processes remains unknown and the exact mechanisms responsible for directing adipogenic cell fate remain to be discovered. As adipose tissue contains a heterogenous population of partially differentiated cells of adipocyte lineage; tissue repair, angiogenesis and neovascularization may be closely linked to the function of ASCs in a complex relationship. This review aims to investigate the link between ASCs and angiogenesis/neovascularization, with references to current studies. The molecular mechanisms of these processes, as well as ASC differentiation and proliferation are described in detail. ASCs may differentiate into endothelial cells during neovascularization; however, recent clinical trials have suggested that ASCs may also stimulate angiogenesis and neovascularization indirectly through the release of paracrine factors.

## 1. Introduction

Adipose-derived Mesenchymal Stem Cells (ASCs) are a medically vital resource for modern regenerative medicine, as they are obtained in a minimally invasive procedure [1]. Adipose depots are found at various locations throughout the body and serve a variety of functions including energy homeostasis of an organism. Subcutaneous adipose tissue obtained from routine surgeries holds therapeutic potential upon reimplantation into the body at sites of injury. Paracrine factors are believed to be responsible, in particular the release of cytokines and growth factors which encourage healing. However, ASCs may also release antioxidants, chaperone proteins, angiogenic and antiapoptotic factors [1,2].

The role of adipose tissue in tissue healing in vivo remains to be fully investigated, in particular the role of ASCs surrounding blood vessels in tissue regeneration. Neovascularization is known to be promoted by paracrine factors released by ASCs implanted to site of injury [2,3] and the chemokines stromal cell-derived factor 1 (SDF-1) and vascular endothelial growth factor (VEGF) have been shown to attract adipose progenitor cells to ischemic sites [1].

ASCs are a multipotent population, differentiating into osteoblast, chondroblast and adipocyte lineages. Notably, in vitro, ASCs have also been shown to differentiate into endothelial and smooth muscle cells, suggesting a possible direct role in neovascularization in vivo. The ability for differentiation of ASCs into Endothelial Cells (ECs) is higher than in Bone Marrow-derived Stem Cells (BMSCs) [2]. Additionally, angiogenesis in developing adipose tissue was shown to be sustainable by recruitment of local ASCs without the need for circulating BMSCs [4].

The potential of ASCs to promote angiogenesis and neovascularization is of key interest when considering myocardial infarction and epicardial adipose tissue. Further research in this area could provide new therapeutic options following ischemic injury. Clinical trials investigating the role of implanted ASCs in promotion of angiogenesis and neovascularization have shown promise in the context of ischemic injury to the myocardium and brain [2,3,5]. In murine model, implantation of preadipocytes promoted angiogenesis, a process which can attract further precursor cells [6,7] and is also required for neovascularization. Vascular ECs may secrete molecules which promote proliferation and differentiation of preadipocytes [7].

Paracrine factors, including angiogenic factors released from ASCs aid in angiogenesis, as well as the recruitment of additional ASCs to sites of angiogenesis and neovascularization, highlighting the interdependent relationship of these components in establishment of new blood vessel networks [8].

ASCs also may serve as supplementary medicine, delivered along with small molecules such as growth factors to implanted bioengineered scaffolds, increasing neovascularization around the site of injury [5]. Other recent technologies developed include methods of inducing neovascularization and angiogenesis by delivery of progenitor cells or small molecules such as growth factors via nanogels [9].

The relative ease of growing, differentiating and dedifferentiating isolated ASCs in vitro culture demonstrates the importance this cell type could play in regenerative medicine, in particular in repair of ischemic injury [10]. Furthering understanding of adipocyte cell lineage and function across stages of maturation in vivo will serve to improve the accuracy of the clinical manipulation and implantation of these cells to injured patients.

## 2. Histological and Cellular Specificity and Plasticity of Adipose Tissue

Adipose tissue, spread throughout the body in depots, serves as the center of the homeostasis of energy in mammals and some other organisms. In humans, it constitutes roughly 20% of body mass in men and 30% in women [11]. As well as functioning as a reservoir for energy supply to various organs, adipose tissue also plays a role in thermogenesis, immune response and the production and secretion of hormones and other small molecules. Adipose tissue is broadly categorized into two main types—white and brown—and further categorized based on physiological location and function as subcutaneous, visceral, marrow, breast and intramuscular fat [12]. Adipocytes are the most common cell type in adipose tissue and are specialized for fat storage. Notably, even fully differentiated adipocyte cells show evidence of stemness and dedifferentiation potential [10]. The stromal vascular fraction in adipose tissue contains cell types such as preadipocytes, fibroblasts, vascular endothelial cells and immune cells.

White adipose tissue (WAT) functions as storage for energy in the form of lipid droplets. These droplets can supply energy to an organism when needed through lipolysis, which involves the breakdown of triacylglycerols into fatty acids and glycerol and is linked closely to insulin resistance and obesity. Excess circulating fatty acids may inhibit insulin function, increase inflammation and pathologically affect secretory properties of adipose tissue [11,13]. As well as triacylglycerol, lipid droplets also store cholesterol esters, with important functions in steroid synthesis and lipid membrane structure. Perilipins are a family of lipid droplet surface proteins important to protection of lipid droplets from the actions of lipases. They are found in all lipid droplets and control access of lipase enzymes to their substrates and thereby regulate lipolysis [14]. The hormones adrenaline and noradrenaline can both activate and inhibit lipolysis and are therefore regarded as master regulators of the process. They interact with adrenergic receptors, a subclass of G-protein coupled receptors and act through the cAMP/Akt pathway to regulate lipolysis. Insulin activates PKB/Akt signaling and thereby counteracts the effects of activatory catecholamines on lipolysis [11,14].

Thermogenesis is a process occurring in brown and beige adipose tissue (BAT) where glucose and fat are burned to produce heat and thereby regulate body temperature. Central to this are the actions of Uncoupling protein 1 (UCP1), an inner mitochondrial membrane protein found in BAT which interrupts the process of ATP synthesis during oxidative phosphorylation by decreasing mitochondrial membrane potential [15]. Differences in morphology of white and brown adipose tissue include a unilocular structure in WAT and a multilocular in BAT. Also present in BAT are numerous and large mitochondria, responsible for the darkened appearance of the tissue. Importantly, BAT is also present in all white adipose depots and has been given the nickname of ‘beige’ adipose tissue. BAT depots also contain a higher degree of neovascularization than WAT [13].

Subcutaneous or close-to-surface depots house most of the large white adipocytes in the body, whereas the majority of small depots are found in visceral depots, surrounding the organs. A distinct type of visceral fat known as epicardial adipose tissue (EAT) covers the heart. This tissue, located on the surface of the myocardium and in close proximity to blood vessels on the heart surface, makes up 20% of human heart mass [16]. It is believed to be important in thermogenesis, metabolic activity and play a mechanically supportive role in preventing warping of the blood vessels by repeated myocardial contraction [17]. Both white and brown adipose tissue have the propensity to secrete adipokines and brown adipokines respectively, such as the hormones leptin and adiponectin, either directly or indirectly through vesicles including exosomes [18].

BAT has been linked to increased expression of the angiogenic factor VEGF in comparison to WAT in a study on rats. Notably, cold exposure further stimulated a temporary and reversible increase of VEGF expression in BAT. This increase in VEGF expression was exclusive to BAT and did not occur elsewhere in the body [19]. The elevated expression of VEGF observed suggests increased proliferation of surrounding endothelial cells and promotion of angiogenesis.

**Figure 1:**
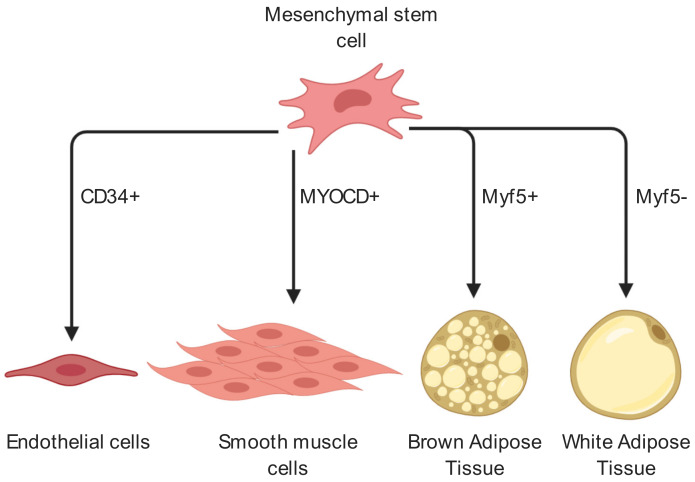
Cell fate of mesenchymal stem cells related to angiogenesis and adipogenesis. MSCs in adipose tissue may differentiate into a variety of cell types including endothelial cells, smooth muscle cells as well as white or brown adipocytes and thereby contribute to angiogenesis and neovascularization. In adipogenesis, Myf5+ and Myf5- cells mark two distinct populations which diverge early in adipogenesis. Myf5+ cells will differentiate into brown adipose tissue while Myf5- will differentiate into white adipose tissue. CD34 is an endothelial cell marker, while MYOCD is a marker for smooth muscle cell differentiation [20,21]. (Created with BioRender).

The origin of the adipocyte cell lineage begins with mesenchymal stem cells (MSCs), which differentiate into adipoblasts, followed by preadipocytes, marking commitment of lineage [6]. If a preadipocyte exits the cell cycle and begins to accumulate fat deposits, it becomes a fully matured adipocyte. Notably, MSCs differentiate early into either Myf5-positive BAT precursors or Myf5-negative WAT precursors [15]. As well as brown and white adipocytes, MSCs show ability to differentiate into both endothelial and smooth muscle cell lines (Figure 1). These multipotent MSCs have long been known to potentially enhance tissue regeneration by implantation to site of injury. It is believed that this process works by the implanted MSCs’ guidance and stimulation of resident stem cells differentiation and the establishment of new blood vessel networks [1]. Importantly, although MSCs have been shown to differentiate into ECs it is likely that following implantation to site of injury, MSCs aid in angiogenesis mostly indirectly, by secretion of factors which promote resident cell differentiation and angiogenesis [1,2,3].

A study profiling the secretome of Adipose-derived MSCs (ASCs) came to the conclusion that human adipose tissue contains a functionally heterogenous population of MSCs, with differing secretory and signaling proteins, multipotency potential and functions in each cell subtype [22]. This serves to highlight the complexity and difficulty of fully understanding adipose tissue function in vivo and achieving succinct sub-cell type classification. The secretome of MSCs, responsible for therapeutic effects following implantation, is composed of paracrine factors; cytokines, RNAs and extracellular proteins such as growth factors [22,23].

The multipotent nature of MSCs makes them an important tool in contemporary regenerative medicine. In particular ASCs, due to high numbers of cells, wide availability of tissue and non-invasive harvesting of cells, are more useful medically than those derived from bone marrow. Digestion of adipose tissue to obtain the SVF is followed by culturing of cells in appropriate medium [24]. In comparison to bone marrow-derived MSCs, those from adipose also show higher long-term stability and retention of stemness in culture and higher proliferation capacity. Notably, support of hematopoiesis by MSCs is more effective by adipose-derived in vivo and in vitro than those derived from bone marrow [25].

In 2006 it was shown that a subpopulation of MSCs derived from BAT could differentiate spontaneously into cardiomyocyte-like cells and improve heart function in rat model. These cardiac progenitor cells were found in significantly higher abundance in BAT than in WAT [26]. Approximately one third of adipose tissue is made up of fully differentiated adipocytes. The remaining two thirds contain large numbers of preadipocytes, which allows adipose tissue to retain plasticity and respond to external stimuli in a variety of ways. Isolated adipocytes grown in culture are able to undergo reversible dedifferentiation into a fibroblast-like phenotype which do not express adipocyte markers such as leptin and GLUT-4 but express markers for osteo- and chondrogenesis: *RUNX2* and *Sox9* and they readily undergo differentiation into various cell types in vitro. Surface antigens expressed were identical between dedifferentiated adipocytes and adipose-derived MSCs. However, unlike MSCs, the dedifferentiated population was highly homogenous, indicating the experimental isolation and profiling of a subset of adipose derived MSCs [10].

Molecules such as insulin, insulin-like growth factor 1 (IGF1), glucocorticoids, mineralocorticoids and thyroid hormones are known to promote differentiation of adipocyte precursors [5,27]. It is well known that blood vessel networks play vital roles in adipogenesis [28]. In murine model, implantation of preadipocytes promoted angiogenesis. Additionally, angiogenesis is required for preadipocyte differentiation, possibly by providing precursors for adipocyte differentiation [6,7], a process which is then further required for neovascularization. Small signaling molecules secreted from vascular ECs in turn promote proliferation and differentiation of preadipocytes [7]. These findings highlight the intricate relationship between adipose tissue function and surrounding vascular networks.

Paracrine signaling constitutes the influence which activated adipocytes have on vascularization and angiogenesis in the immediate blood capillary environment, mediated through molecules such as leptin, angiopoietins, HGF, GM-CSF, VEGF, FGF-2 and TGF-β. Adipose tissue-derived MSCs also possess the ability to increase neovascularization directly through differentiation into ECs [10].

## 3. Molecular Mechanisms Regulating Growth and Proliferation of Adipocytes

Molecular mechanisms regulating the formation of adipose tissue became the target of numerous studies and clinical trials due to their potential application in diagnosis, treatment and prevention of diabetes, dyslipidemia, obesity and many metabolic diseases. Adipocyte turnover, either in humans or rodents, is a dynamic process depending on several factors, including nutritional cues, environmental stimuli or lifestyle choices, affecting cellular composition of adipose tissue [29]. Understanding adipogenesis requires integration of animal studies, clinical trials and analysis of molecular mechanisms involved in adipose stem cell niche. Transcriptional control of adipocytes’ growth is regulated by genes influencing preadipocyte formation, such as *Zfp4, Krox20, Elk1, Med23, Bmi1* and genes regulating the proliferation of adipocyte precursor cells, ASCs and MSCs, including *E2F4*, *RBL2*, *KLFs 4, 5, 6, 11, 15* and *16*. Additional transcriptional factors involved in these mechanisms are positive key transcriptional effectors, like C/EBPα, C/EBPβ, C/EBPδ, AP-1, E2Fs, PPARγ, PRRX1, STAT5A and negative transcriptional effectors, being GATAs, PREF-1, Wnt-10b, Wnt-5a. Moreover, hepatocyte growth factor (HGF), vascular endothelial growth factor (VEGF), platelet-derived growth factor receptor beta (PDGFRB), fibroblast growth factor 21 (FGF21) are strictly involved in the growth and proliferation of adipocytes, whose molecular markers are generally CD29, CD140a, CD140b. Furthermore, cell cycle proteins crucial during adipocyte development include cyclin D, cAMP-response element binding protein (CREB), cyclin-dependent kinase inhibitors, including p21, p27^KIP and^ hormones, such as estrogen, insulin, somatomedin C (IGF-1) [30,31,32,33,34,35].

The absence of *Zfp4, Med23, Elk1 or Krox20* inhibits early genomic responses to signaling cascades responsible for adipogenesis [32]. Further validation of the epistasis pathway through knockdown of these genes and genes belonging to the *KLFs* family resulted in inhibition of adipocytes proliferation. However, insulin-induced adipogenesis is restored by Krox20, connecting adipogenesis and insulin signaling pathways [32]. The downregulation of Med23, gene product of *Elk1,* also binds strongly the *Krox20* promoter [32]. *Krox20* fully expressed in association with crucial pro-adipogenic transcription factors CCAAT/enhancer-binding proteins (C/EBPα, C/EBPβ and C/EBPδ) is bound by pocket proteins (Rbs) [36].

**Figure 2:**
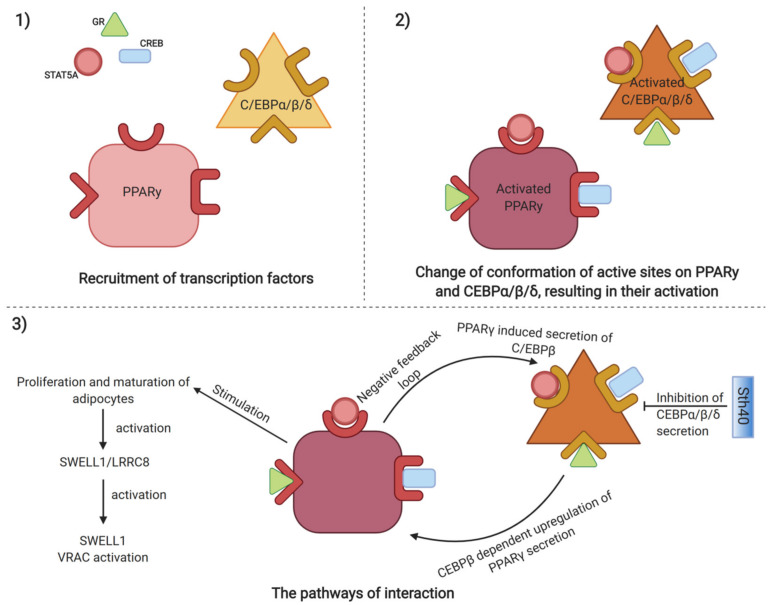
Mechanisms regulating adipose stem cells expansion, including activation of target CCAAT/enhancer-binding proteins and PPARγ through recruitment of transcription factors including activators of transcription CREB, GR and STAT5, resulting in change of conformation of active sites on target proteins. Activated target CCAAT/enhancer-binding proteins regulate PPARγ through Sth40 mediated inhibition of C/EBPβ, while PPARγ itself stimulates expansion of adipose stem cells, by SWELL1/LRCC8 mediated activation of SWELL1 mediated VRAC signaling pathway. (Created with BioRender).

Peroxisome proliferator-activated receptor γ (PPARγ) transcriptional signaling cascade, acting in adipose progenitor cells (APCs), is crucial for adipose stem cell niche expansion, regulating tissue homeostasis and repair [31]. Two stages of establishment of the PPARγ transcriptional network are distinguished. At first, groups of transcription factors are recruited, including an activator of the glucocorticoid receptor (GR), a signal transducer, an activator of transcription 5A (STAT5A) and CREB activates PPARγ and CCAAT/enhancer-binding proteins [37]. C/EBPβ is then bound by pocket proteins (Rbs) and the complex C/EBPβ–Rbs further upregulates PPARγ, which, in turn, either regulates the secretion of C/EBPβ through a negative feedback loop or induces the proliferation and maturation of adipocytes (Figure 2) [38]. Additionally, both C/EBPβ and C/EBPδ are controlled at translational level by serine/threonine kinase 40 (Stk40) [39]. Stk40 represses the levels of C/EBP proteins and the knock-out of Stk40-KO cells leads to increased levels of C/EBP proteins and promotes differentiation pathways into embryonic fibroblasts. Interestingly, the knockdown of C/EBPβ downregulates adipogenic differentiation in *Stk40*-KO cells. A recent study found a leucine-rich repeat containing transmembrane protein complex SWELL1-/LRRC8 to be activated following proliferation of adipose tissue mass, further regulated in adipocytes and mechanical adipocyte swelling, predisposing to SWELL1-mediated VRAC activation [40].

Early stages of cell proliferation are regulated and the Wnt/β-catenin signaling controls the homeostasis of the mature tissue [41]. The role of Wnt signaling in adipocyte lineage function and differentiation is still not fully understood, however the canonical β-catenin pathway is believed to be a key regulator of adipocyte maturation. Whether β-catenin binds subsequently to CREB binding protein, promoting cell potency maintenance or to p300, resulting in differentiation of precursors, remains to be fully investigated [42]. β-catenin may have a stimulatory function on adipocytes’ proliferation by upregulating the secretion of adipokine from already differentiated adipocytes [42]. Recent studies discovered that adipogenesis may have a potential therapeutic effect on EMT-derived breast cancer cells, disconnecting the oncogenic cells from invasive phenotype through EMT/MET transcription factors and TGF-β signaling [43]. Li et al. proposed that, when overexpressed, novel transcription factors, including MAFF, MXD4 and BATF3, showed capacity to suppress proliferation and adipogenesis of human ASCs [44]. *Suresh* and *West* hypothesized and confirmed, that 3D culture of ASCs yields better differentiation potential compared to 2D standardized culture [45]. A recent study concerning the process of adipogenesis in the context of hematopoietic stem cell niche proposed de-repression of *Pax3* gene as a method to rescue the functional knockout of *Bmi1*, depleting the inhibitory effect of *Bmi1* on adipogenesis and promotion of adipocytic differentiation [46].

## 4. External Stimuli Regulating Proliferation of Adipocytes

Adipocytes growth and ASCs are studied in obese animal models to understand the mechanisms underlying impaired and rapid proliferation of adipose tissue mass. Two processes are involved in uncontrolled adipocyte proliferation, including hyperplasia, characterized by elevated formation of adipocytes from preadipocytes and hypertrophy, characterized by increased adipose size. Moreover, insulin-like growth factor I (IGF-1), prostaglandins or fatty acids are naturally derived stimulators of adipogenesis, while growth hormones, cytokines and transforming growth factor-β (TGF-β) are inhibitors [47]. Elevated expression of galectin-3 is observed in mice following a high fat diet, in comparison with controls [48]. Moreover, berberine inhibits mouse primary preadipocytes isolated from epididymal white adipose tissue, reducing the expression of galectin-3 promoter [49]. An increase in cell proliferation of preadipocytes was associated with obesity, contradicting the previous results obtained by Nadler et al. [50]. In contrast, ASCs harvested under hypoxia showed increased levels of growth factor VEGF, which, in turn, stimulated proliferation of adipocytes, improving the wound healing properties of ASCs [51]. *Moringa oleifera* leaf extract also has anti-proliferative activity and inhibits adipogenesis in 3T3-L1 adipocytes by suppressing common adipogenesis markers, including PPARγ, FABP4, cEBPβ and ADIPOR1 [52]. The miRs expression profiles related to PPARγ dependent adipogenesis are interconnected with the upregulation of miR-425. miR-425 itself regulates the expression of *Mapk14*, leading to high-fat diet-induced obesity [53]. Information on the functional significance of the of internal and external molecular regulators were summarized in Table 1.

A recently published study showed that dehydrodiconiferyl alcohol (DHCA), extracted from the stems of *Cucurbita moschata,* significantly reduced obesity in obese mice models by inhibiting the expression of adipocyte markers, including CCAAT/enhancer-binding proteins and peroxisome proliferator-activated receptor γ [54]. Interestingly, another study found fresh *Panax ginseng* leaves, in association with spherical gold nanoparticles, to have suppressive effects on adipogenesis, simultaneously downregulating PPARγ/CEBPα signaling in 3T3-L1 mature adipocytes [55]. Boron was also found to inhibit adipogenesis in progenitor cells, by regulating; the β-catenin and AKT in Wnt/β-catenin pathways, the expression of adipogenesis related proteins, including PPARγ and cEBPα and regulation of cell cycle genes [56]. Wei et al. demonstrated that the gradually decreasing age-dependent expression of the Hedgehog interacting protein, responsible for the interaction with hedgehog family and the modulation of hedgehog signaling, is involved in the down-regulation of the expression of Cyclin B, Cyclin D and Cyclin E, suppressing preadipocyte proliferation and increasing the expression of Glut4 and PPARγ [57]. Apart from inhibiting interactions between cells in ovarian cancer, the secreted protein, acidic and rich in cysteine (SPARC) has been investigated in terms of communication between omental adipocytes, showing that SPARC may inhibit in vivo metabolic programming of adipocytes, including reduced adipocyte-induced homing and proliferation [58]. In summary, environmental stimuli including diet, disease state, disease activity and naturally derived regulatory chemicals, potentially interplay with transcriptional control and play significant roles in molecular mechanisms regulating the proliferation of adipocytes and ADSCs (Table 2).

## 5. Differentiation and Transdifferentiation of Adipocytes

A major goal for ameliorating diabetes and obesity is understanding the molecular biology underlying the differentiation of white and brown adipocytes to enhance sensitivity to insulin [59]. Even though current studies are largely employing mice, these models show similarity to human tissue in terms of vascularization and differentiated adipose tissue. Functionally distinct types of adipocytes were identified; white adipocytes that store energy in the form of triglycerides and brown adipocytes that work in association with beige adipocytes to metabolize lipids for heat generation, the former has recently been described to interplay with adipocytes called thermogenic [60]. Beige adipocytes are distinguished among brown adipocytes population by the expression of UCP1 marker, classified as dormant beige or active beige [61]. Shao et al. suggested two molecular pathways to acquire beige adipocyte phenotype, reprogramming mature white adipocytes to brown adipocytes UCP1+ or de novo differentiation from preadipocytes. While insulin signaling occurs in the white adipose tissue (WAT), which itself accounts for the majority of adipose tissue, non-shivering thermogenesis occurs in brown adipose tissue, marked by expression of uncoupling protein-1 (UCP1), a marker of terminal differentiation [62]. Among white adipocytes there are three different subpopulations, including Wilms’ tumor 1+ adipocyte precursor, transgelin+ adipocyte precursor and myxovirus 1+ adipocyte precursor, distinguished by different gene expression profiles, growth hormones and insulin sensitivity [63]. Paracrine signal fibroblast growth factor 8b (FGF8b) induces UCP1 expression in epididymal white preadipocytes through intracellular pathway connecting FGF8b and the UCP1 promoter, interfering with adipogenesis [64]. Moreover, β2 adrenergic stimulation holds anti-inflammatory effect in high-fat diet-induced obesity [65]. Also fibroblast growth factor 21 (FGF21) has anti-inflammatory properties in obesity related inflammation and is observed to increase sensitivity to insulin [66]. Secretion of FGF21, in association with mono(2-ethylhexyl)phthalate (MEHP) and lactate, upregulates the expression of *Fgf21* and facilitates glucose uptake in the MEHP-treated adipocytes [67]. FGF21 further facilitates the so called ‘browning’ of WAT through the transdifferentiation of white adipocytes to brown adipocytes by UCP-1 dependent and independent mechanisms, leading to elevated noradrenaline levels and thermogenesis [68]. The adipocyte maturation derived transcription factors, CEBPA and PPARG, responsible for regulation of downstream genes, are reduced upon the administration of growth differentiation factor 11 (GDF11) that activates TGF-β/Smad signaling pathway [69]. White adipocytes also hold capacity to transdifferentiate into beige adipocytes, if exposed to cold stress, chronic PPARγ induction or β3 adrenergic activation [38]. Transdifferentiation is here defined as the capacity of white adipocytes to differentiate into other mature adipocyte cells (e.g., beige or brown, respectively), omitting the intermediate pluripotent state.

In undifferentiated 3T3-L1 preadipocytes the concentration of insulin receptors is halved compared to the IGF-1 receptors, however, after insulin induction the concentration of insulin receptors increases and adipocytes start to respond to the hormone [70]. Selenium-binding protein 1 (SELENBP1), a recently discovered marker of mature adipocytes regulating the oxidation of methanethiol, is reported to be upregulated during terminal differentiation of 3T3-L1 preadipocytes [71]. While treatment of hemangioma stem cells from infantile hemangiomas with IGF-1 positively influenced adipogenesis and expression of PPARγ, inhibition of IGF-1 receptor caused suppression of signals from C/EBPα, C/EBPβ and PPARγ [72]. In vitro cultivated preadipocytes cell lines 3T3-L1 and 3T3-F442A re-entered mitotic clonal expansion and differentiated into fully matured adipocytes when stimulated with IGF-1 arrested growth [73]. IGF-1 receptor works in association with lipoprotein receptor-related protein (LRP1), which increases the affinity of Src homology 2/α-collagen (ShcA) and acts as its docking site. This also binds to the IGF-1 receptor-LRP1 complex, which in turn recruits growth factor receptor-bound protein 2 (Grb2), that binds ShcA and stimulates the Ras/MAP kinase pathway [74]. Interestingly, the differentiation of 3T3-L1 preadipocytes to mature adipocytes is inhibited by placental extract in the beginning of adipogenesis [75].

## 6. External Stimuli Regulating Differentiation of Adipocytes

Fully differentiated adipocytes require stimulation of precursor cells with different factors, for example the effects of biochemical cues of adipose specific extracellular matrix (ECM) not only enhance adipogenic differentiation but they also stimulate adipogenesis in ASCs [76,77]. Osteopontin (OPN) is downregulated in obese individuals and its silencing promotes ASCs adipogenic differentiation [78]. Precursors of adipocytes are able to differentiate into brown adipocytes when treated with norepinephrine, which increased lipolysis and *Ucp1* mRNA expression [79]. Interestingly, a single small-molecule compound—RepSox—is able to induce the browning of white adipocytes or, alternatively, induce brown tissue adipogenesis inhibiting TGF-β receptor [80]. Furthermore, Tu et al. reported Tranilast, LY2157299 and A83-01 to hold a similar inhibitory effect to the RepSox. Not only does Galectin-3 induce proliferation of adipocytes but it plays an important role in maturation and differentiation of preadipocytes to lipid-laden adipocytes [49]. Moreover, neural tissue derived Neuropeptide Y (NPY) mRNA is upregulated in obese Zucker rats of elevated visceral adiposity [81]. Yang et al. demonstrated that increased levels of NPY protein stimulated primary preadipocytes of rats, while simultaneously increasing visceral adiposity. In contrast, DeltaFosB transcription factor (ΔFosB) inhibits adipogenesis [82]. Rowe et al. studied ΔFosB expression in terms of regulatory function in insulin sensitivity, including possibility to target ΔFosB for the treatment of obesity. Apart from the inhibitory effect of ΔFosB transcription factor on adipogenesis, ΔFosB also downregulates the expression of early adipocyte differentiation markers [82]. Adipogenic differentiation is also promoted by the miR-30a inhibition [83]. Similarly, inhibition of miR-30a restored functional knockdown of *H19 {80}.* One of the prostaglandins, prostacyclin acting on the prostacyclin receptor is found to have pro-adipogenic effects not only on precursor cells but also on mature adipocytes [84]. *Pseudomonas aeruginosa*-derived pyocyanin not only played a part in chronic adipose cachexia but also contributes to reduction in body mass through suppression of adipocyte differentiation [85].

Impairment to the recruitment of signal transducer and activator of transcription 3 (STAT3) upon its phosphorylation due to mutation of *LepRb Tyr 1138* results in promotion of adiposity [86]. Furthermore, knockout of the acetyl CoA acyltransferase 2 in *ACAA2-KO* preadipocytes resulted in downregulated expression of *PPARγ* and lipoproteinlipase *(LPL),* leading to the inhibition of adipocyte differentiation pathways [87]. Autophagy that contributes to metabolic processes and differentiation pathways, if stimulated with leptin, results in the development of adipose tissue hypoxia, which leads to induced insulin resistance [88]. Functional experiments aiming to improve the quality of meat through identification of molecular processes responsible for formation of intramuscular fat characterized as a quality indicator of the flavor and tenderness of chicken meat, identified gga-miR-18b-3p to inhibit differentiation of intramuscular adipocytes by targeting the 3′UTR of *Acot13* gene [89]. In vitro studies showed that collagen I promotes adipocytogenesis in ASCs [90]. miR-130b-5p and hsa-miR-23a-5p are demonstrated to induce adipogenic differentiation of human adipose tissue-derived stromal stem cells (hASCs) [91]. Guo and Cao further speculated about the cytokine interleukin-1α (IL-1α) involvement in suppression of adipogenic proliferation and differentiation. Overexpressed miR-107 is established to inhibit adipocyte differentiation by downregulation of *Notch3* signaling, affecting in turn the expression of CDK6 [92].

## 7. Molecular Mechanisms of Angiogenesis and Neovascularization

The capillary system is essential for providing oxygen and nutrients to cells, whilst also transporting carbon dioxide and waste. Components of the system are made by the processes of angiogenesis and neovascularization. Although the two processes are similar in their roles of forming new blood vessels, angiogenesis is defined as building on new branches or protrusions from already formed vessels. Neovascularization, on the other hand, is the formation of entirely new blood vessels. In adulthood, neovascularization does not tend to occur unless induced by ischemia [93] or following an arterial blockage. For improving clinical therapies, it is important to understand the molecular mechanisms of neovascularization and angiogenesis.

To learn about the mechanisms of blood vessel formation, zebrafish and postnatal retinal material are used, as animal studies undergoing healing are inaccessible [93,94]. However, the rat vascular balloon injury model is another method that can showcase neovascularization [95]. The chick chorioallantoic membrane (CAM) model is also used to study neoangiogenesis [96].

Tip cells gain information from the extracellular environment and act as sensors that will mediate cell proliferation and migration [97]. Tip cells are involved in both neovascularization and angiogenesis. Proliferating stalk cells and paracrine effectors, such as granulocyte-colony stimulating factor (G-CSF), which helps recruit stem cells, help to form the structures [98,99].

Examples of chemotaxins that may attract progenitor cells to invade ischemia sites [98] include the chemokines stromal cell-derived factor 1 (SDF-1) and vascular endothelial growth factor (VEGF) genes that have shown attractive properties. Another example of chemokines that attract progenitor cells are immune competent cells, such as MCP-1 or IL-8 [100]. It has been suggested that plaque angiogenesis is induced by ischemic conditions which cause noticeably larger plaque sizes after the regeneration of endothelial cell populations [98]. Delivery vehicles that transport progenitor endothelial cells or growth factors such as heparin-Pluronic (HP) nanogels could be used to stimulate angiogenesis or neovascularization [9].

Due to the specialized physiology and shape of organs, neovascularization and angiogenesis are thought to tailor new capillary formation [101]. Endothelial progenitor cells with endothelial markers such as CD34, CD133 and ATP-binding cassette subfamily G member 2 (ABCG2) are driven to differentiate into an endothelial phenotype in areas where oxygen perfusion is low to restore homeostasis [98,102,103,104]. CD34^+^ adult stem cell populations are also known to be located in bone marrow [105]; however, there are non-bone marrow sources of endothelial progenitor cells such as tissue resident stem cells or vessel wall-derived endothelial cells [98]. To correctly identify the origin of stem cell populations used in the body to undergo neovascularization and angiogenesis, strategies such as fate mapping studies could set apart the population markers [98]. During blood vessel formation, myeloid stem cells have been linked to the muscle repair process [98]. Ex vivo, these stem cells express both CD14^+^ and CD34^−^ endothelial markers together. Additionally, myeloid stem cells were shown to be part of the vessel assemblage in the study done by Urbich et al. It is thought that myeloid cells are intermediates to mature hematopoietic stem cells, which links them to their role of muscle regeneration [98]. The hox proline-rich homeodomain gene (*Prh*) and serine/threonine kinases PIM-1 (*pim-1* oncogene), which is induced by VEGF activity, aid in the differentiation of endothelial progenitors.

## 8. Angiogenesis

Angiogenesis is potentially a guided process regulated by growth factors and cell signals [101]. Tip cells in the trachea have sensors that carry the signals to start the process of angiogenesis. The tracheal tip cells are receptive to guidance cues from signals to move. To receive such signals and act accordingly, these tip cells have dynamic filopodia. Meanwhile, specialized endothelial cells (endothelial sprouts) are thought to also have filopodia on their tips that signal cell migration and guidance. In a study done by Gerhardt et al. [101], they found endothelial sprouts extend filopodia in response to extracellular VEGF-A signaling. Transplanted endothelial cells can be used to start the process of angiogenesis [106]. VEGFR2 helps regulate cell migration. Also, cells in the vascular stalk were influenced by VEGF-A to proliferate once the growth factor reached its receptor and VEGFR2 regulates this by using the same receptor [107]. Therefore, the processes of stalk cells proliferating and endothelial sprouts migrating are in opposition since they are regulated by the same growth factors. A delicate growth factor balance must be achieved to mediate successful angiogenesis. Over 30 miRNAs upregulate or downregulate angiogenesis[108,109] and mRNAs may have downstream effects that affect angiogenesis, including the increased expression of VEGF. In particular, miR-126 and miR-132 are two miRNAs that are well understood and involved in the proliferation and migration of endothelial cells for blood vessel formation [110].

**Figure 3:**
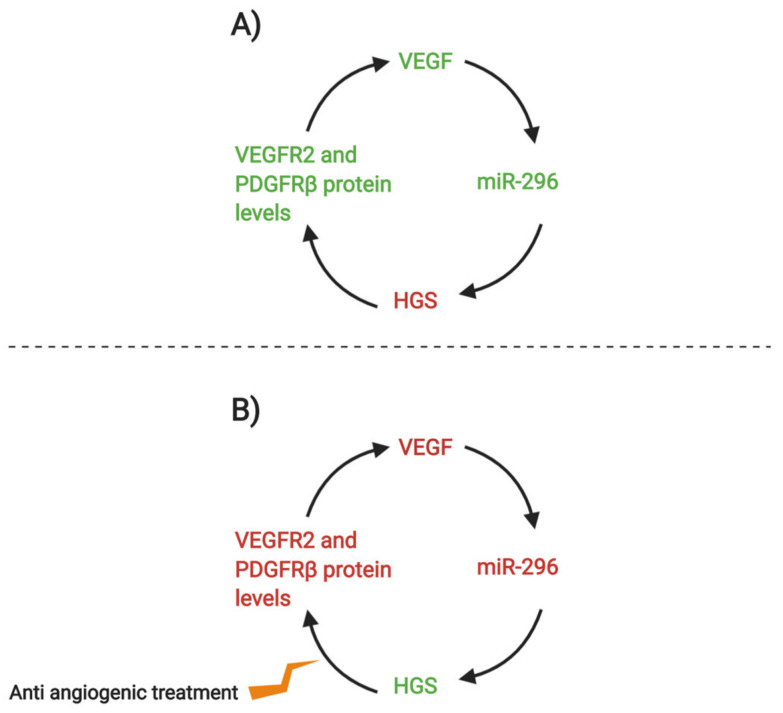
(**A**) Vascular endothelial growth factor (VEGF) stimulates non-coding miR-296, which then lowers the expression of platelet-derived growth factor (HGS). HGS is responsible for controlling the degradative sorting of VEGFR and platelet-derived growth factor receptor (PDGFR), so their protein expression levels are regulated. However, since miR-296 lowers HGS, then VEGF2 and PDGFRβ levels increase, stimulating VEGF activity. (**B**) Anti-angiogenic treatment is used to downregulate the activity of VEGFR and PDGFR, which decreases their protein levels, which could combat the effects of the miR-296 cycle [113]. (Created with BioRender).

Tumor tissue can induce formation of blood vessels in a process called neoangiogenesis [111]. Tumors that are distant from blood vessels undergo oxidative stress. To supply more oxygen to the deficient cells, new blood vessel protrusions and outgrowths restore cell homeostasis and it is associated with cancer cell migration. Treatments which could prevent neoangiogenesis include nitric oxide to inhibit proliferation or anti-VEGF agents [99,112], VEGFR and PDGFR are common targets in these therapies [113], especially in combination with other cancer treatments such as chemotherapy [114]. miR-296 has been associated with neoangiogenesis [113]. Below is an example of a potential anti-angiogenic treatment to decrease the activity of miR-296 (Figure 3).

Angiogenesis is induced by the gene expression of ASCs; however, ASCs have the downside of also increasing the risk of cancer development by their production of specific growth factors and cytokines [115]. For example, a study compared the effect on breast cancer progression of c-KIT+ ASCs and other subpopulations of ASCs [116]. The expression of c-KIT in endothelial progenitor cells stimulates proliferation, differentiation and cell survival and the results of the study suggests c-KIT caused a larger growth of tumors due to promoting interleukin-3. Another pro-inflammatory cytokine associated with tumor growth is interleukin-6 [117,118], which in the case of melanoma drives cancer progression as an autocrine stimulator [119,120,121]. Additionally, a study by Chang et al. suggests that measuring the serum levels of interleukin-6, alongside interleukin-1β and tumor necrosis factor α can be used to monitor cancer progression, as the proinflammatory cytokines are expressed more intensely in later stages of disease [122].

IL-8 overexpression in ASCs, whilst stimulating angiogenesis and thereby improving microvessel density, was also shown to increase tumor growth in melanoma and lung metastasis [123]. The same study also conducted an IL-8 knock-out, which resulted in decreased cell survival and tumor growth. Tumor growth can be facilitated by IL-8, by the protein breaking down the extra cellular matrix (ECM) in a way that supports cancer progression [124,125]. Matrix metalloproteinases such as MMP-2 and MMP-9 are induced by IL-8 and other angiogenic growth factors such as SDF-1 and VEGF, to allow bi-directional communication between melanoma cells and ASCs [125,126,127].

Overall, paracrine factors enable the construction of tumor network structures which provide oxygen and nutrients, therefore leading to further progression of cancer. Furthermore, ASCs are recruited by the tumor microenvironment by cytokine/receptor pairs to promote tumor growth. However, tumor growth can also be inhibited by the injection of proinflammatory cytokine interleukin-15 into adipose tissue via recruitment of natural killer cells to inhibit tumor metastasis [128]. This suggests ASCs can have both favorable and unfavorable effects in angiogenesis and cancer growth.

## 9. Neovascularization

When neovascularization activity is high it is associated with the risk of diabetic retinopathy and tumor progression [93,129], highlighting the importance of mechanisms to regulate the process. VEGF-A helps encourages cell proliferation, survival and migration for neovascularization [101].

Neovascularization may be a spontaneous process and the formation of new primary vessels are followed by a branch regression [101]. The survival of endothelial cells might influence the structure of the blood vessel. In turn, VEGF and surrounding signals will impact the endothelial cells. However, Prakash et al. believe neovascularization is a controlled process, at least in the central nervous system [130].

To understand the neovascularization pathway, Manavski et al. used histochemistry to measure VE-cadherin levels and endothelial cell clonal expansion [93]. Microvessels are known to be formed with the presence of VE-cadherin, which can be considered a marker for mature endothelial cells and affects cell–cell adhesion [104]. The cadherin levels increased after ischemia, suggesting that the hypoxic event drove cell maturation. Furthermore, endothelial cells can then be involved in neovascularization. However, the cadherin levels increased sporadically and momentarily, which supports the theory of paracrine stimulation.

## 10. Possible Relationship between Adipose-Derived Stem Cells and Neovascularization and Angiogenesis Processes—Recent Trials and Potential Clinical Applicability

ASCs have been deeply studied as a source of stem cells for the treatment of multiple conditions. Easily harvested, ASCs show paracrine activity and exhibit differentiation potential towards different cell lineages (adipogenic, osteogenic, chondrogenic and myogenic lineages). Moreover, as adipocytes seem to have the same progenitor as endothelial cells, ASCs also participate in the formation of neo-vascular like structures. Therefore, ASCs may influence the growth of capillary networks necessary in adipose tissue enlargement [131]. In addition, ASCs show great potential in regenerative medicine providing immunosuppressive properties and low immunogenicity [132].

By enhancing angiogenesis and vasculogenesis, ASCs promote neovascularization, which is fundamental in the treatment of post ischemic injuries. In fact, isolated from the stromal vascular factor of human adipose tissue, ASCs expressing CD34, CD133 and ABCG2 were found to differentiate into endothelial cells and to contribute to the revascularization of the ischemic hindlimb in mice [133]. Not only do ASCs stimulate angiogenesis through differentiation into epithelial cells but also through paracrine activity are proven to release angiogenic factors. Non-adipocyte stromal cells in the fat tissue were observed to secrete multiple angiogenic and antiapoptotic growth factors which are critical to the promotion of angiogenesis, such as VEGF and HGS but also P1GF, bFGF, angiogenin, GM-CSF, MCP-1 and SDF-1α [134,135].

Therapeutic angiogenesis could be beneficial not only in the case of myocardial infarction, ASCs can similarly help other conditions, like peripheral vascular disease, ischemic diseases (such as ischemic injury, ischemic heart disease, ischemic heart disease, ischemic cerebral disease and peripheral ischemic vascular disease), acute tubular necrosis, diabetic retinopathy and traumatic spinal cord injuries [136,137,138,139].

## 11. Ischemic Heart Disease

Adequate angiogenesis increases oxygen and nutrient supplies to tissues and thus preserve their function, impaired in ischemic diseases. One of the major causes of death and hospitalization worldwide is myocardial infarction. Different therapies have been suggested as a substitute to heart transplantation and the delivery of autologous cells as a treatment for people with cardiovascular diseases has shown favorable outcomes. The benefit of implementing ASC as a therapy for heart conditions is due not only to their ability to differentiate into cardiomyocytes but also their paracrine activity; through the release of antiapoptotic factors, they promote the maintenance of pre-existing cardiac cells [134]. Autologous ASCs administered, in a porcine model of myocardial infarction through intracoronary injection, increased angiogenesis showing better cardiac function and lesser unfavorable ventricular remodeling [140]. ASCs differentiated into epithelial cells. Many other animal models showed similar beneficial effects in coronary artery diseases where ASCs exhibited differentiation ability towards cardiomyocytes [141]. Multiple studies suggested that the enhancement of angiogenesis and vasculogenesis, in cardiovascular environments, are not only due to the differentiation potential of ASCs but also to their paracrine effects [142,143].

Moreover, myocardial regeneration can be promoted by inducing myogenic and angiogenic mechanisms. In fact, by cellular cardiomyoplasty, which relies on the implantation of cells including ASCs in order to promote tissue growth and angiogenesis, the size of infarct area can be reduced. Cellular cardiomyoplasty limits post-ischemic remodeling and restores the regional myocardial contractility [144,145,146]. The first cellular cardiomyoplasty achieved with ACCs was performed in 2007 and it proved how the promotion of angiogenesis and cardio protection via this type of stem cell was a great viable clinical option [140]. As a matter of fact, in the myocardium there is an absence or very limited number of stem cells and implementing them showed positive results.

Furthermore, multiple studies showed the occurrence of cells in adipose tissue that could differentiate into endothelium, also improving blood flow, capillary density and perfusion. A clinical trial showed an improvement in coronary perfusion. The administration of ASCs by intracoronary infusion in patients with acute myocardial infarction lead to a reduction of the infarct size and an enhancement in cardiac cells size [147]. Moreover, trans-endocardial injection of autologous ASCs to patients with ischemic cardiomyopathy showed beneficial results in cardiac performance, myocardial perfusion and exercise capacity [148]. MyStromalCell Trial used for the treatment of chronic IHD ASCs and pre-treated with VEGF-A165, proved a safe option for promoting cell survival [149].

## 12. Ischemic Cerebral Diseases

Ischemic stroke causes a reduction in the oxygen supply, leading to an increase in angiogenesis to meet greater metabolic requirements. Considering recovery after stroke, a possible treatment focuses on increasing blood vessel density leading to reduced morbidity and longer lifespan of the subject. ASCs may be a safe solution to limit the damage of the brain infarct and improve neurological function [150]. Moreover, the reduction of the infarction area upon ASC administration is related to an increase in neurogenesis and vasculogenesis [151].

Different studies reported that delivering ASCs upon stroke in mice lead to behavioral recovery. The promotion of angiogenesis is restricted to the area of the infarct and is connected with the elimination of necrotic brain tissue, while vascularization is fundamental for striatal neurogenesis [152]. The administration of hASC-conditioned medium into lateral ventricle resulted in a reduction of infarction volume and lesser neural cell apoptosis, together with greater epithelial cells proliferation and micro vessels density [153]. Moreover, administering ASC rats models with middle cerebral artery occlusion stimulates the protection of cerebral function, the reduction of brain cell death and the promotion of angiogenesis and neurogenesis [154]. ASCs transplanting can promote angiogenesis and revascularization in rat brain after focal cerebral ischemia enhancing TGF-β1 expression in the brain [155]. Moreover, administration of xenogeneic and allogeneic ASCs show both no side effect and an equal effect on recovery [156].

## 13. Ischemic Limb Disease

Administering ASC to mice with ischemic limb disease supports increased blood flow and capillary density [136]. Human ASCs (hASCs) were also administered to a mouse model of ischemic limb disease increasing capillary density via paracrine activity enhancing muscle recovery [157]. Heparin/protamine micro/nanoparticles, biodegradable carriers for ASCs usually employed in therapies aiming at promote angiogenesis, appeared to be novel optimal treatments to reverse limb ischemia in mouse models [158].

By inducing specific gene expression patterns in a precise and well-controlled manner, it is possible to improve the therapeutic angiogenic effects of ASCs. For example, inducing the expression of v-myc in hASC leads to greater proliferation and migration potential and greater secretion of VEGF [159]. Moreover, transducing hASC with VEGF165 and transplanting it into a murine ischemic hindlimb model contributes to an increase in angiogenesis and revascularization [160].

## 14. Allograft

Other applications of ASCs exploit their ability to increase angiogenesis towards the development of treatments of other conditions, such as ischemic colitis, ischemic colonic anastomoses and ischemic kidney injury [161,162,163]. These properties of ASCs are used to increase the vascularization of allotransplantations, increasing tolerance and reducing toxicity. Preclinical and clinical settings of ASC showed auspicious therapies, showing immunotolerant properties and easy isolation. As autologous fat transplantation is restricted by low vascularization, fat absorption and fibrosis, co-transplantation of ASC-derived extracellular vesicles with fat tissue into a mouse model increased tissue volume retention due to vascularization enhancement and inflammatory response [164,165]. The paracrine products of ASCs, analyzed by loading them into a microencapsulation device, enhanced the angiogenic activity of endothelial cells and influenced macrophages secretion. The results showed increased vascularization and decreased fibrotic tissue formation [132]. Biodegradable scaffolds provide a great technology in soft tissue regeneration for the delivery of ASCs, which easily differentiate into adipose tissue. Platelet rich plasma work synergistically with ASCs to increase the biocompatibility of the biodegradable scaffolds [166]. Further research on the optimal scaffold maximizing soft tissue augmentation is still needed. Moreover, ASCs were observed to interact with endothelial cells, altering, under hypoxia, the levels of interleukin-6, interleukin-8, monocyte chemoattractant protein-1 and vascular endothelial growth factor. These effects underpin a beneficial role of the interaction between these two types of cells in soft tissue healing, once again proving the potentiality of ASCs regulation of inflammation and angiogenesis [167]. An alternative to stem cell transplantation therapy is the employment of stem cell-conditioned medium (CM), obtained from ASCs 3D-cultures rich in angiogenic and/or antiapoptotic factors, such as vascular endothelial cell growth factor, fibroblast growth factor 2, hepatocyte growth factor and chemokine (C-X-C motif) ligand 12. CM showed positive results in ischemic region in mice, increasing endothelial cells growth [168].

In the field of implants, ASCs application has also been tested to replace tissue upon trauma, cancer or congenital diseases. The implementation of ASCs and argon on synthetic biomaterials with argon plasma surface modifications ensured the enhancement of vascularization of tissue engineered constructs. Argon plasma surface modification promotes ASCs-related increase of angiogenesis, improving the survival rate of large tissue implants [169]. The survival of fat grafting is observed to be enhanced by co-transplantation of ASC-derived exosomes, responsible for proangiogenic potential of stem cells. This technique not only promotes graft survival, it also increases neovascularization and decreases inflammation [170].

Moreover, Manavella et al. showed that neovascularization can be enhanced in grafted ovarian tissue following the secretion of VEGF and ASCs differentiation into vessels. ASCs are a significant advance in the improvement of the lifespan and quality of ovarian tissue and their effects should be considered further for clinical application [171]. Further studies of the ovarian tissue cryopreservation and transplantation demonstrated faster and better reoxygenation and revascularization of the graft, which means increased follicle survival and reduced apoptosis [172].

In conclusion, the implementation of ASCs exploiting their characteristic ability to promote and enhance angiogenesis and stimulate neovascularization in a multitude of tissues is beneficial in the treatment of numerous conditions and diseases. Looking into techniques to fully exploit these tendencies, such CO2 laser on ASCs which activates redox pathways increasing cell proliferation and enhances the secretion of angiogenic molecules, may further expand the range of clinical applications [173].

## Figures and Tables

**Table 1:** Functional significance of internal and external molecular regulators of adipocyte proliferation including varying impact of biological and chemical reagents. The effector function of a particular molecule, such as inhibition or stimulation is determined and supplemented accordingly by the following mechanisms and signaling pathways.

Authors of Research	Molecules/Family of Molecules/Plant Species	Effector Function	Mechanism	References
Chen and Wang	β-catenin	Stimulation	Adipokine upregulation	[8]
Li et al.	MAFF, MXD4, BATF3	Inhibition	Overexpression of *Maff, Mxd4*, *Batf3*	[43]
Hu et al.	BMI1	Inhibition	Repression of *Pax3*	[45]
Ali et al.	IGF-1, prostaglandins, fatty acids	Stimulation	Extracellular signaling	[46]
Ali et al.	Growth hormones, cytokines, TGF-β	Inhibition	Extracellular signaling	[46]
Doğan et al.	Boron	Inhibition	Inhibition of PPARγ, CEBPα Regulation of β-catenin, AKT	[55]

**Table 2:** Functional significance of including varying impact of external herbal effectors on adipocyte proliferation. The function of a particular effector, such as inhibition or stimulation is determined and supplemented accordingly by the following mechanisms and signaling pathways.

Authors of Research	Molecules/Family of Molecules/Plant Species	Effector Function	Mechanism	References
Wang et al.	Berberine	Inhibition	Destabilization of Gal-3 mRNA, resulting in decrease of Gal-3 promoter activity	[48]
Lee et al.	Hypoxia	Stimulation	Upregulation of vascular endothelial growth factor (VEGF) and basic fibroblast growth factor (bFGF)	[50]
Balusamy et al.	*Moringa oleifera*	Inhibition	Inhibition of PPARγ, FABP4, cEBPβ, ADIPOR1	[51]
Lee et al.	dehydrodiconiferyl alcohol	Inhibition	Inhibition of C/EBPα, C/EBPβ, C/EBPδ, PPARγ	[52]
Simu et al.	*Panax ginseng*	Inhibition	Inhibition of PPARγ, CEBPα	[54]

## Abbreviations

ABCG2	ATP-binding cassette subfamily G member 2
ASC	Adipose-derived mesenchymal stem cell
BAT	Brown adipose tissue
CAM	Chick chorioallantoic membrane
EAT	Epicardial adipose tissue
G-CSF	Granulocyte-colony stimulating factor
hASC	Human adipose-derived mesenchymal stem cell
HGS	Hepatocyte growth factor-regulated tyrosine kinase substrate
HP	Heparin-Pluronic
IL-8	Interleukin 8
MSC	Mesenchymal stem cell
PPARγ	Peroxisome proliferator-activated receptor γ
PDGFR	Platelet-derived growth factor receptor
pim-1	Serine/threonine kinases PIM-1
Prh	Proline-rich homeodomain gene
SDF-1	Stromal cell-derived factor 1
VEGF	Vascular endothelial growth factor
VEGFR	Vascular endothelial growth factor receptor
WAT	White adipose tissue
